# Isolation, Identification, and Characterization of One Degradation Product in Ambroxol by HPLC-Hyphenated Techniques

**DOI:** 10.3797/scipharm.1310-21

**Published:** 2014-01-12

**Authors:** Veera Raghava Raju Thummala, Mrutyunjaya Rao Ivaturi, Someswara Rao Nittala

**Affiliations:** 1 Analytical Research and Development, Integrated Product Development, Dr. Reddy’s Laboratories Ltd., Bachupally, Hyderabad-500 072, India.; 2 School of Chemistry, Andhra University, Visakhapatnam-530003, A.P., India.

**Keywords:** Ambroxol, RP-HPLC, Unknown impurity, NMR, LC-MS/MS

## Abstract

This study details the isolation, identification, and characterization of ambroxol’s unknown impurity. One unknown impurity of ambroxol was formed in the formulated drug under stress conditions [40°C /75% relative humidity (RH) for 6 months] with the relative retention time (RRT) 0.68 in RP-HPLC. The impurity was enriched by exposing it to heat and it was isolated by using preparative HPLC. The enriched impurity was purified and characterized using the following sophisticated techniques: 2D NMR (gDQ-COSY, gHSQC, and gHMBC), FTIR, and LC-MS/MS. On the basis of the spectral data, the impurity was characterized as *trans*-4-(6,8-dibromoquinazolin-3(4*H*)-yl)cyclohexanol.

## Introduction

**Fig. 1:**
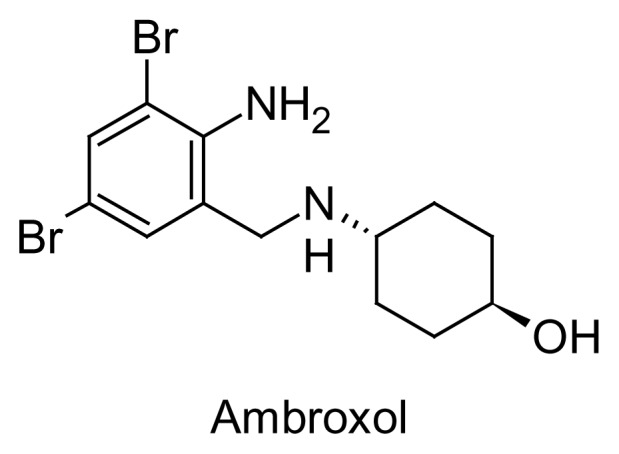
Structure of Ambroxol

Ambroxol hydrochloride, chemically *trans*-4-[(2-amino-3,5-dibromobenzyl)amino]cyclo-hexanol hydrochloride [1], is a semi-synthetic derivative of vasicine from the Indian shrub “Adhatoda vasica”. It is an expectoration improver and a mucolytic agent used in the treatment of bronchial asthma and chronic bronchitis. Ambroxol hydrochloride has also been reported to have cough-suppressing effects and anti-inflammatory action. Recently, the inhibition of nitric oxide-dependent activation of soluble guanylate cyclase was suggested to be one of the molecular mechanisms of therapeutic action of ambroxol hydrochloride, also used in pulmonary alveolar proteinosis in pulmonary distress and infant respiratory distress syndrome. The molecular structure is shown in Fig. 1.

In general, solid active pharmaceutical ingredients (APIs) are formulated with excipients as tablets, syrup and/or capsules. Since the active ingredient interacts with the excipients and the formulated product is stored at different conditions, the study of API stability is critical in the drug development process. Many factors can affect the stability of a pharmaceutical product, some of them include the stability of the active ingredient, the manufacturing process, the environmental conditions (such as heat, light, and moisture during storage), as well as some chemical reactions such as oxidation, reduction, hydrolysis, and racemization that might occur [2, 3]. The study of stability under stressed conditions is important since it can cause many degradation reactions.

**Fig. 2:**
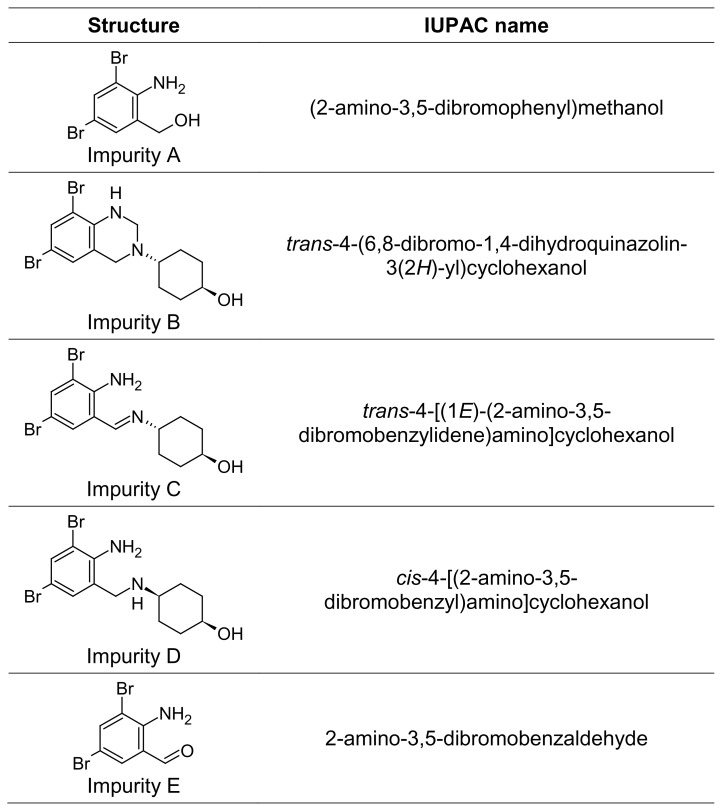
Structures of ambroxol’s known impurities

Several spectrophotometric methods have been reported for the qualitative and quantitative determination of AMB from pharmaceutical formulations [6–9]. Various other methods such as HPLC [10–13], GLC [14, 15], the sequential injection technique coupled with a monolithic column [16], LC-MS [17], capillary electrophoretic [18] and fluorescence detection [19] are also reported for its determination from biological fluids. A few of the degradants and other impurities of ambroxol reported in the British Pharmacopiea [5] are shown in Fig. 2. One unknown impurity was observed during the stability study of ambroxol syrup. This impurity was observed to be at more than the identification threshold as per ICH guidelines [4]. None of the literature methods discussed the identification of unknown impurities during stability studies of ambroxol.

The present article deals with the identification and characterization of one unknown degradant impurity formed during the storage of the drug product at stressed conditions [40°C/75% relative humidity RH] for 6 months. This impurity was isolated by preparative HPLC and the structure was confirmed using FTIR, LC-MS/MS, and NMR spectroscopy.

HPLC-hyphenated techniques are now widely used for the structure elucidation of trace amounts of the degradation products without a complicated isolation process. LC–MS/MS has been one of the powerful techniques for the identification of small quantities of drug degradation products [20]. In the present study, the impurity was isolated by preparative HPLC and characterized by FTIR, NMR, and LC-MS/MS.

## Experimental

### Chemicals, Reagents, and Samples

Ambroxol syrup was received from the formulation research and development laboratory of Dr. Reddy’s Laboratories Ltd., IPDO, Hyderabad, India. Diammonium hydrogen phosphate was procured from Merck, Germany. HPLC grade acetonitrile, methanol, and orthophosphoric acid were purchased from Merck, Germany. High purity water was prepared by using the Millipore Milli-Q plus purification system.

### Chromatographic Conditions for HPLC

HPLC measurements were carried out using a reversed-phase Waters Symmetry C18, 250 × 4.6 mm, 5 μ particle size column operated at 25°C with gradient elution at 0.8 mL min^−1^ using the mobile phase buffer 0.01 M diammonium hydrogen phosphate of pH 7.0 (pH-adjusted with dilute orthophosphoric acid solution); UV absorbance at 248 nm; injection volume 10 μL. Mobile phase A consisted of a pH 7.0 phosphate buffer and acetonitrile (80:20 v/v); mobile phase B consisted of a pH 7.0 phosphate buffer, acetonitrile, and methanol (20:75:5 v/v/v). The LC gradient program was set as: time (min)/% mobile phase B: 0.01/25, 45/60, 50/75, 55/75, 60/25, and 65/25. A mixture of water and acetonitrile (50:50 v/v) was used as diluent for the sample preparation.

### Isolation of the Unknown Impurity by Preparative HPLC

#### Enriching the Unknown Impurity

Ambroxol syrup was exposed to heat at 105°C for 5 days. The stressed syrup equivalent to 1000 mg of ambroxol was transferred into a 50-ml volumetric flask. 30 ml of diluent was added and the sample was sonicated for 30 minutes with intermediate shaking. It was then made up to volume with diluent (20 mg/ml). This solution was injected into the liquid chromatography. The observed degradation was 10.35%.

#### Chromatographic Conditions for Preparative HPLC and Preparative Isolation

Preparative HPLC was performed using a reversed-phase Inertsil C18 column (250_20mm i.d., 5 mm, Zodiac Silica Company) on the Agilent Preparative HPLC system. Mobile phase A consisted of water and acetonitrile in the ratio of 80:20 (v/v), respectively. Mobile phase B consisted of acetonitrile and water in the ratio of 80:20 (v/v), respectively. Preparative HPLC was carried out at a flow rate of 15 mL/min, the column oven temperature was maintained at ambient conditions, and the Rheodyne injector was used for injecting the samples into the chromatographic system. The gradient program for Impurity I followed was time (min)/% mobile phase B: 0/5, 12/5, 16/85, 32/85, 36/5, and 40/5). Peak cut criteria for the isolated impurity was set based on the peak retention time. Fractions >95% purity were pooled together and concentrated by the Rotavapor to remove solvents, then lyophilized using freeze drying to obtain a pure compound with greater than 98% purity.

### NMR

The ^1^H and ^13^C NMR data for ambroxol’s unknown impurity was recorded in CDCl_3_ at 500 MHz and 125 MHz, respectively, on the Varian Unity Innova 500 MHz spectrometer. The chemical shift values were reported on the δ scale in ppm with respect to TMS (δ = 0 ppm) and CDCl_3_ (δ = 77 ppm) as internal standards, respectively. Also DEPT, gDQ-COSY, gHSQC, and gHMBC experiments were performed in CDCl3.

### FTIR

The FTIR spectrum of ambroxol’s unknown impurity was recorded on the Perkin Elmer model spectrum series FTIR as KBR pellets.

### Mass Spectrometer

The ESI mass spectrum of ambroxol’s unknown impurity was recorded on the 4000-Q-Trap LC-MS/MS system. The sample was introduced into the system through HPLC by bypassing the column.

## Results and Discussion

The purpose of this work was to study the stability of ambroxol syrup under stressed conditions. A small quantity of syrup was kept at 40°C /75% RH in stability chambers for about 6 months.

The initial purity and that after the stressed conditions were studied by HPLC. The chromatogram revealed one unknown impurity formed during the accelerated stress conditions. The unknown impurity was eluted at the relative retention time (RRT) 0.68 which was referred to as Impurity I. Impurity I was isolated by preparative HPLC and the structure was identified by spectroscopic techniques (IR, NMR, and LC-MS/MS).

### FTIR

**Fig. 3:**
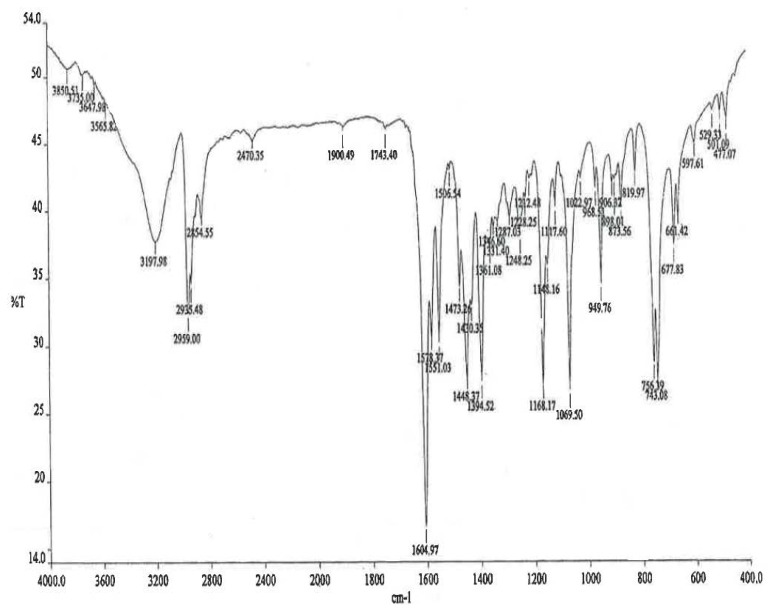
FTIR spectrum of ambroxol’s unknown impurity

The FTIR spectrum of the ambroxol impurity is shown in Fig. 3. The assignments are mentioned in Table 1.

### NMR Study

**Fig. 4:**
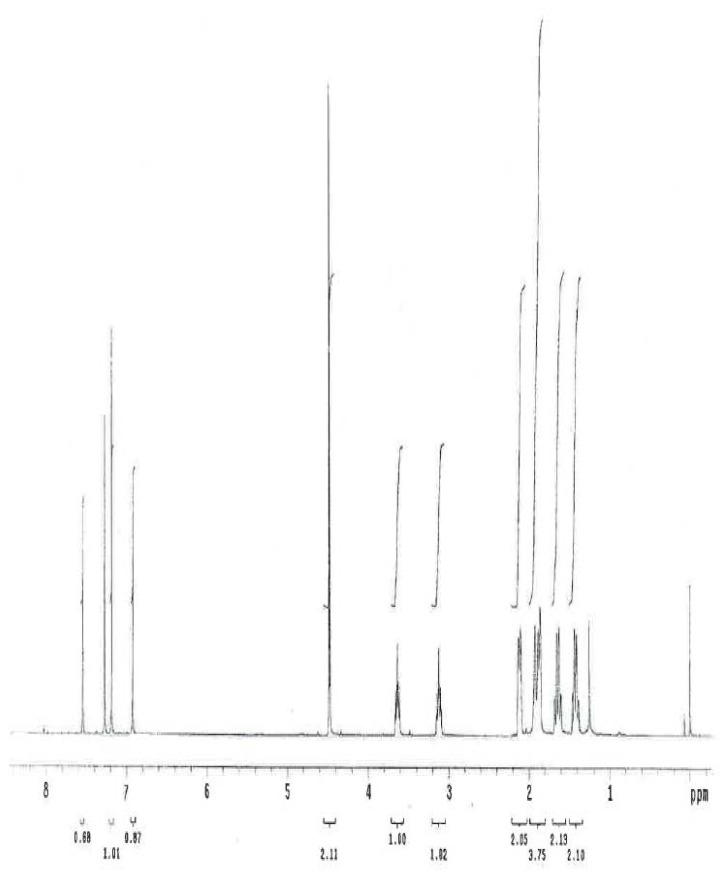
^1^H NMR spectrum of ambroxol’s unknown impurity in CDCl_3_

**Fig. 5:**
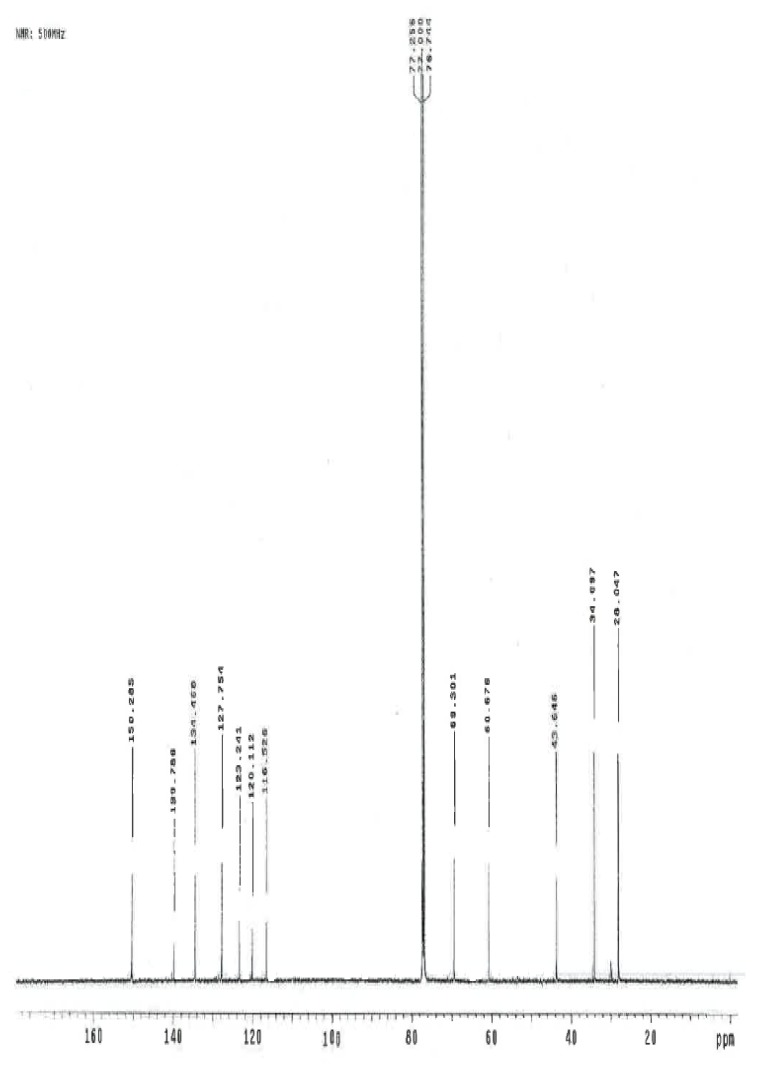
^13^C NMR spectrum of ambroxol’s unknown impurity in CDCl_3_

**Fig. 6:**
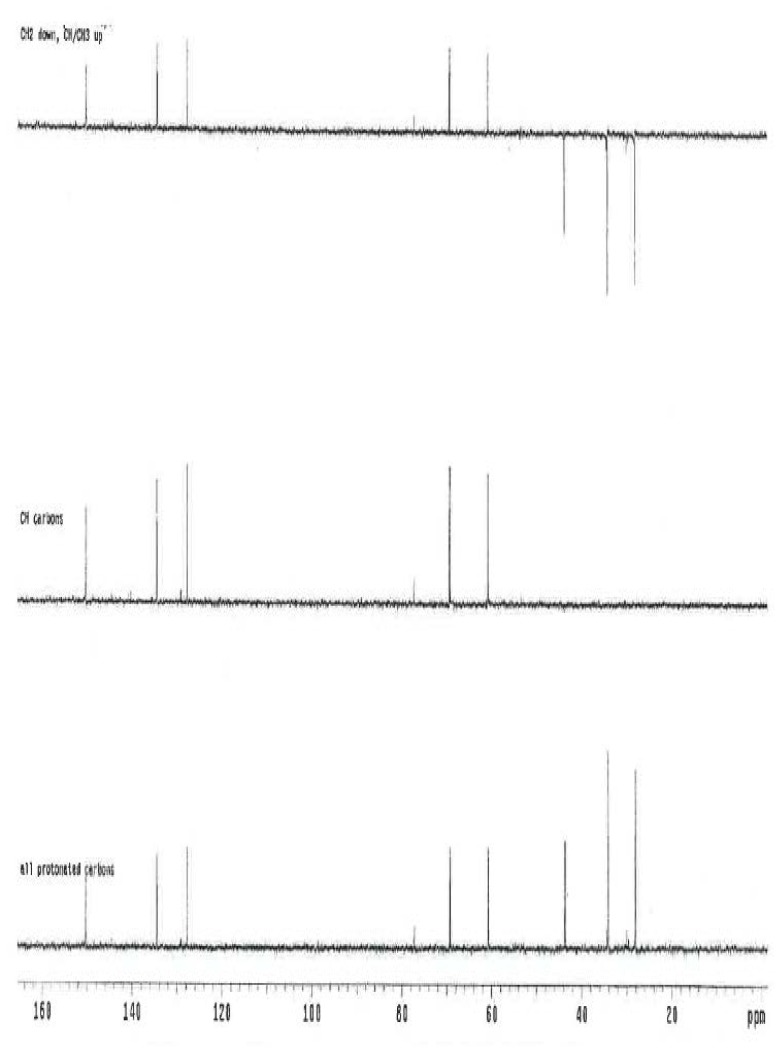
DEPT spectrum of ambroxol’s unknown impurity in CDCl_3_

**Fig. 7:**
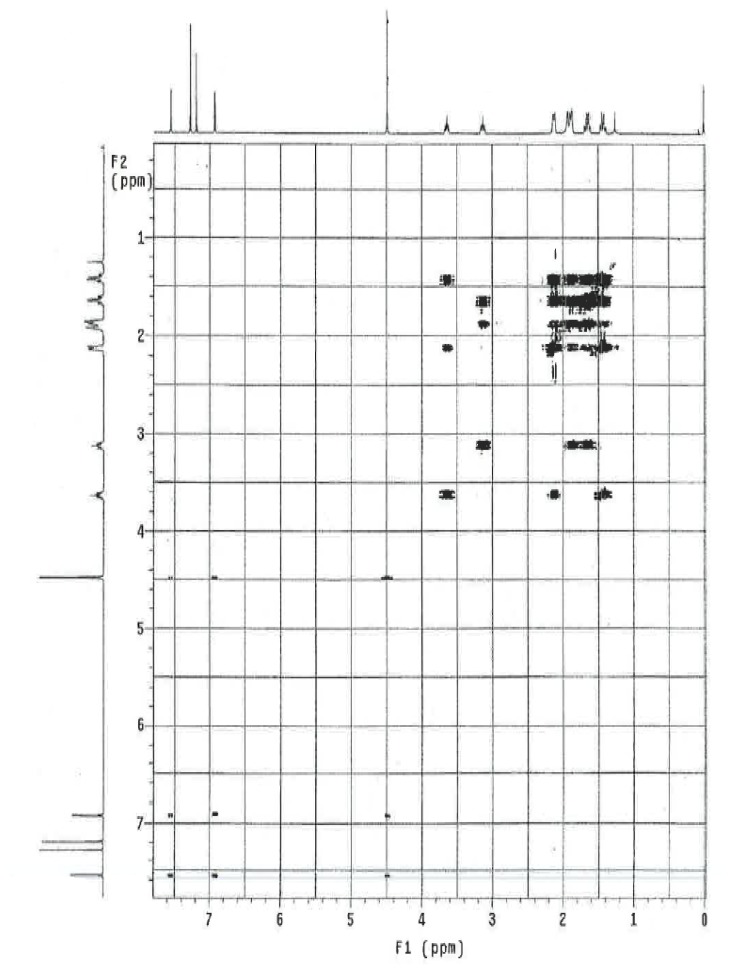
gDQ-COSY spectrum of ambroxol’s unknown impurity in CDCl_3_

**Fig. 8:**
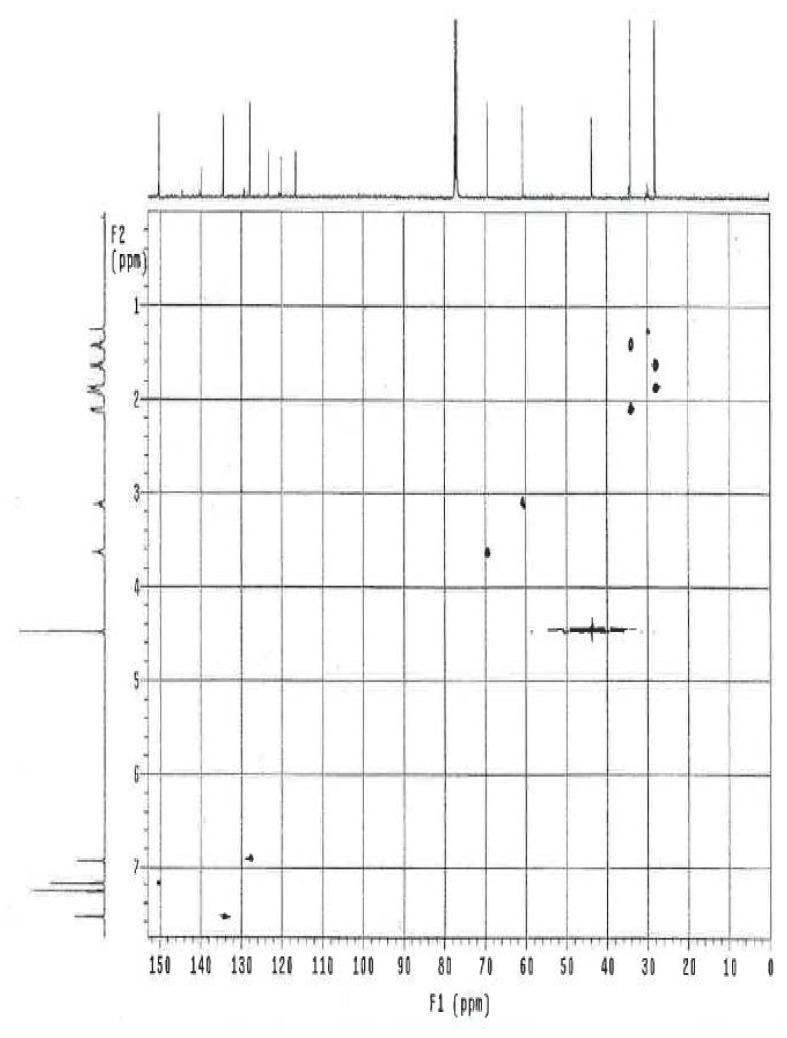
gHSQC spectrum of ambroxol’s unknown impurity in CDCl_3_

**Fig. 9:**
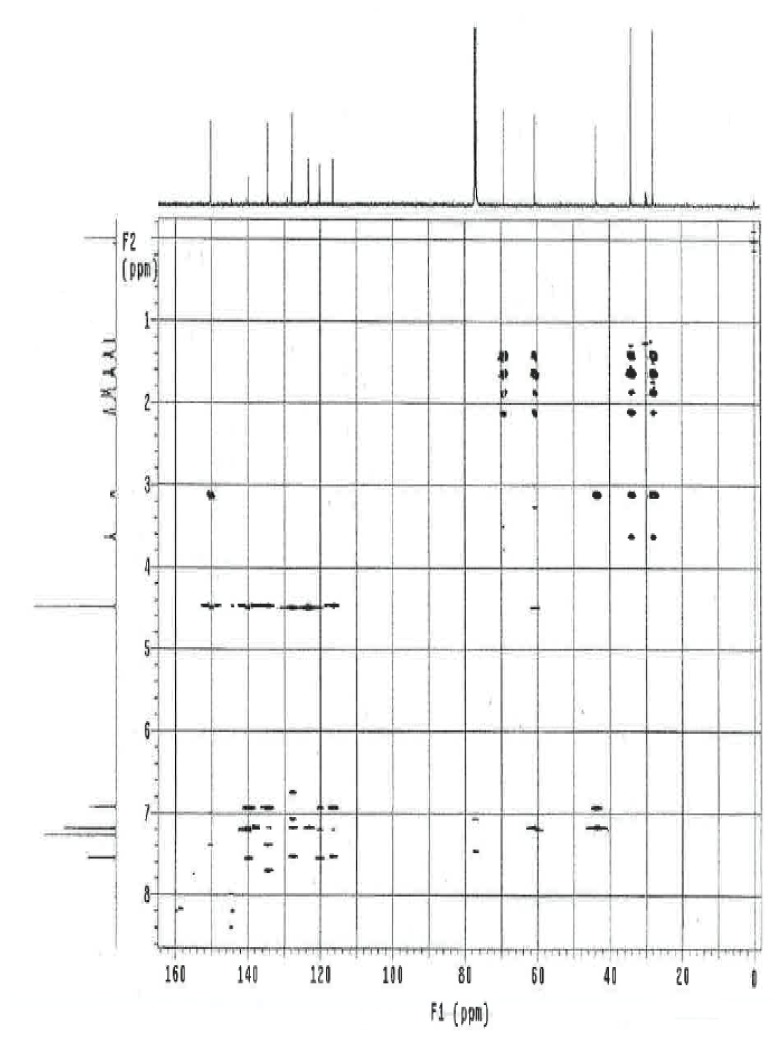
gHMBC spectrum of ambroxol’s unknown impurity in CDCl_3_

**Fig. 10:**
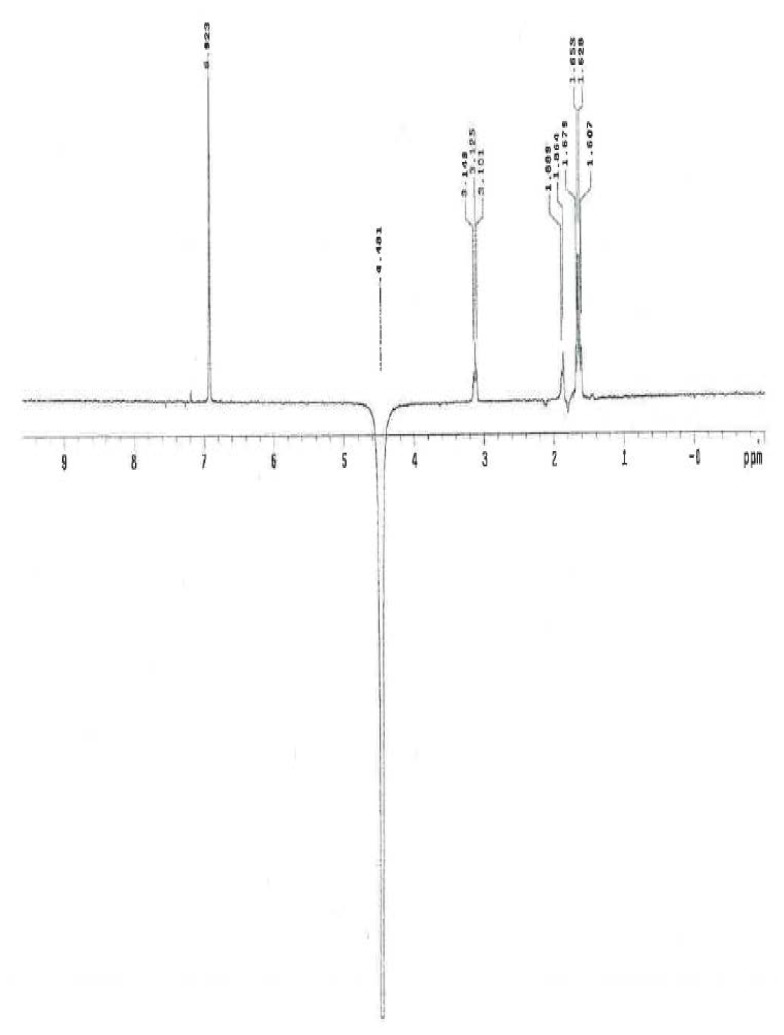
ID nOe spectrum of ambroxol’s unknown impurity in CDCl_3_

The ^1^H and ^13^C NMR spectrum of ambroxol’s unknown impurity are shown in Fig. 4 & 5. The DEPT experiment revealed the presence of methine groups as positive peaks, while methylene showed as negative peaks which is displayed in Fig. 6. The GDQ-COSY and gHSQC spectra (Fig. 7 & 8) helped to identify the 1H-1H and 1H-13C correlations. The gHMBC spectrum helped in assigning the quaternary carbons (Fig. 9). In the ID nOe experiment (Fig. 10), irradiated methylene protons at 4.48 ppm (position 10) showed the connectivity through space with the protons at 6.92 and 3.12 ppm (Position 7 and 11, respectively). This confirmed the double bond formation at position 2. The NMR assignments are given in Table 2.

### Mass Spectral Data

**Fig. 11:**
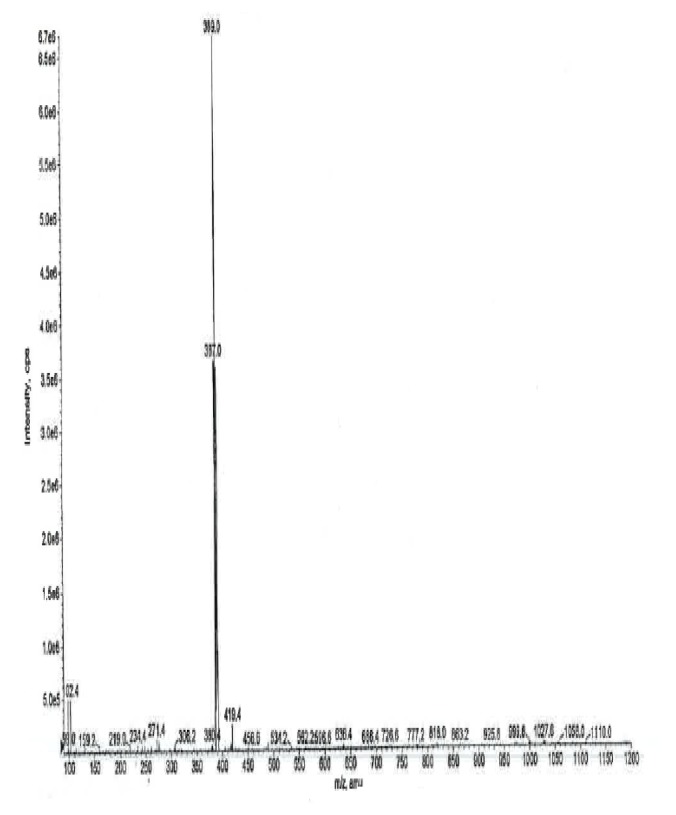
The ESI +ve mass spectrum of ambroxol’s unknown impurity

**Fig. 12:**
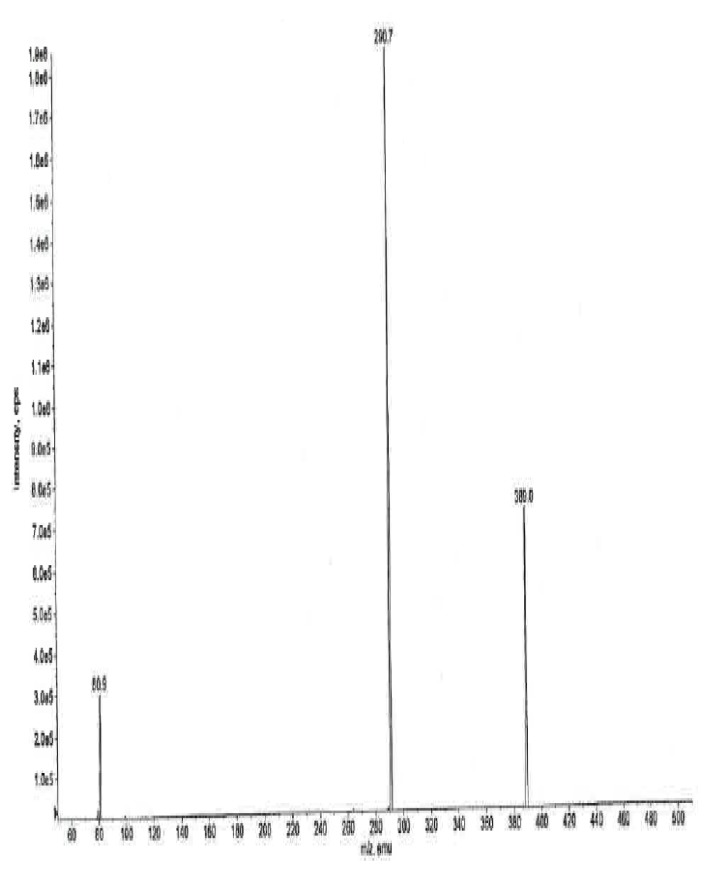
The LC/MS/MS mass spectrum of ambroxol’s unknown impurity

**Fig. 13:**
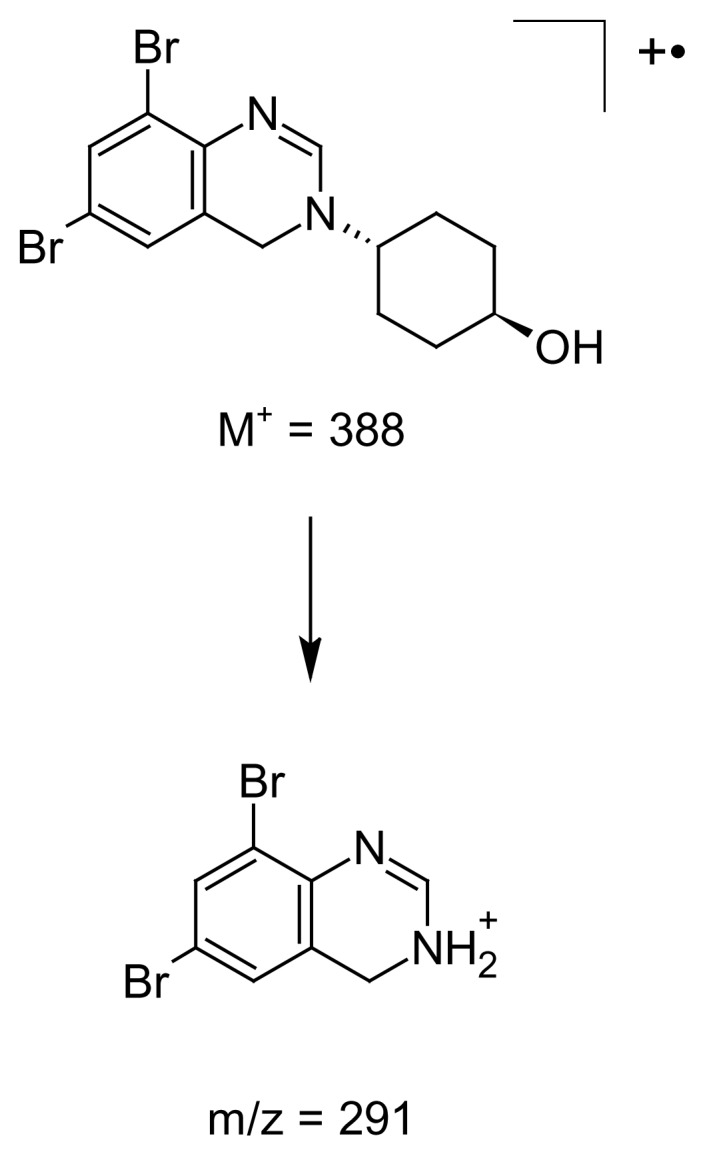
Plausible fragmentation pattern for the unknown impurity from the LC-MS/MS spectrum

The ESI +ve ionization spectrum (Fig. 11) data of ambroxol’s unknown impurity displayed the protonated molecular ion [M + H]+ at m/z 389 corresponding to the molecular formula C_14_H_16_Br_2_N_2_O. The LC-MS/MS scan (Fig. 12) confirmed the molecular formula. The plausible fragmentation pattern for the unknown impurity based on the LC-MS/MS scan is shown in Fig. 13.

**Fig. 14:**
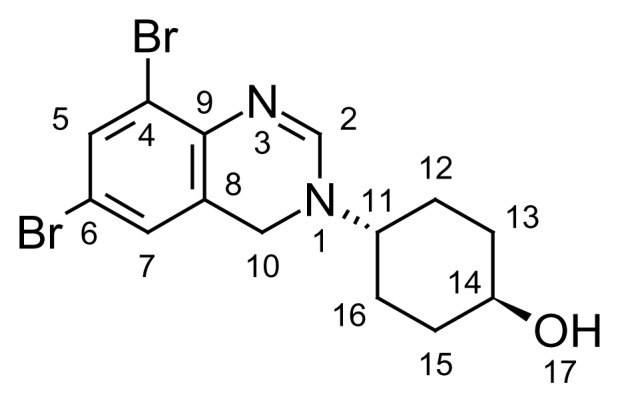
Structure of ambroxol’s unknown impurity

From the IR, NMR, and mass spectral data, the structure of ambroxol’s unknown impurity was confirmed as *trans*-4-(6,8-dibromoquinazolin-3(4*H*)-yl)cyclohexanol. The elucidated structure is shown in Fig. 14.

## Conclusion

One unknown impurity in ambroxol syrup was generated during the accelerated stressed conditions. This impurity was isolated by preparative HPLC and characterized as *trans*-4-(6,8-dibromoquinazolin-3(4*H*)-yl)cyclohexanol by IR, NMR, and LC-MS/MS. Such advanced methods will be utilized for structure elucidation of impurities of other active ingredients.

## Figures and Tables

**Tab. 1:** IR assignments of ambroxol’s unknown impurity

Wave Number (cm^−1^)	Assignment	Mode of vibration
3198	**-O-H**	Stretching
2959, 2935, 2854	Aliphatic **–C-H**	Stretching
1605, 1551	Aromatic **-C=C**	Stretching
1448, 1394	Aliphatic **–C-H**	Bending
1168	**-C-O**	Stretching
1069	**-C-N**	Stretching
756, 743	Aromatic **–C-H**	Bending

**Tab. 2:** NMR assignments of ambroxol’s unknown impurity

Atom^1^	^1^H	δ (ppm)	J(Hz)^2^	^13^C	COSY	HSQC	DEPT
2	1H	7.19	s	150.3	–	(2H, 7.19)	CH
4	–	–	–	120.1	–	–	–
5	1H	7.55	d, 2.5	134.5	–	(5H, 7.55)	CH
6	–	–	–	116.5	–	–	–
7	1H	6.92	d, 2.5	127.7	–	(7H, 6.92)	CH
8	–	–	–	123.2	–	–	–
9	–	–	–	139.8	–	–	–
10	2H	4.48	s	43.6	–	(10H, 4.48)	CH_2_
11	1H	3.12	m	60.7	(12, 16Ha, 1.64) (12, 16He, 1.88)	(11H, 3.12)	CH
12, 16	2Ha	1.64	dq,	28.0	(12, 16He, 1.88) (13, 15Ha, 1.42) (11H, 3.12)	(12, 16Ha, 1.64)	CH_2_
	2He	1.88	m	–	(12, 16Ha, 1.64) (11H, 3.12)	(12, 16He, 1.88)	–
13, 15	2Ha	1.42	dq, 12.5	34.1	(13, 15He, 2.12) (12, 16Ha, 1.64) (14H, 3.64)	(13, 15Ha, 1.42)	CH_2_
	2He	2.12	d, 12.5	–	(13, 15Ha, 1.42) (14H, 3.64)	(13, 15He, 2.12)	–
14	1H	3.64	m	69.3	(13, 15Ha, 1.42) (13, 15He, 2.12)	(14H, 3.64)	CH

1 Refer to the structural formula in Figure 14 for numbering;

2 This column gives the 1H-1H multiplicity and coupling constants;

s…singlet, d…doublet, q…quartet, m…multiplet.
